# Bioactive Compounds Found in Brazilian Cerrado Fruits

**DOI:** 10.3390/ijms161023760

**Published:** 2015-10-09

**Authors:** Elisa Flávia Luiz Cardoso Bailão, Ivano Alessandro Devilla, Edemilson Cardoso da Conceição, Leonardo Luiz Borges

**Affiliations:** 1 Câmpus Henrique Santillo, Universidade Estadual de Goiás, Anápolis, Goiás CEP 75132-903, Brasil; E-Mails: elisaflavia@gmail.com (E.F.L.C.B.); ivano.devilla@gmail.com (I.A.D.); 2 Laboratório de Pesquisa em Produtos Naturais, Faculdade de Farmácia, Universidade Federal de Goiás, Goiânia, Goiás CEP 74605-170, Brasil; E-Mail: ecardosoufg@gmail.com; 3 Escola de Ciências Médicas, Farmacêuticas e Biomédicas, Pontifícia Universidade Católica de Goiás, Goiânia, Goiás CEP 74605-010, Brasil

**Keywords:** Brazilian savanna, functional foods, phenolic compounds, secondary metabolites

## Abstract

Functional foods include any natural product that presents health-promoting effects, thereby reducing the risk of chronic diseases. Cerrado fruits are considered a source of bioactive substances, mainly phenolic compounds, making them important functional foods. Despite this, the losses of natural vegetation in the Cerrado are progressive. Hence, the knowledge propagation about the importance of the species found in Cerrado could contribute to the preservation of this biome. This review provides information about Cerrado fruits and highlights the structures and pharmacologic potential of functional compounds found in these fruits. Compounds detected in *Caryocar brasiliense* Camb. (pequi), *Dipteryx alata* Vog. (baru), *Eugenia dysenterica* DC. (cagaita), *Eugenia uniflora* L. (pitanga), *Genipa americana* L. (jenipapo), *Hancornia speciosa* Gomes (mangaba), *Mauritia flexuosa* L.f. (buriti), *Myrciaria cauliflora* (DC) Berg (jabuticaba), *Psidium* *guajava* L. (goiaba), *Psidium* spp. (araçá), *Solanum lycocarpum* St. Hill (lobeira), *Spondias mombin* L. (cajá), *Annona crassiflora* Mart. (araticum), among others are reported here.

## 1. Introduction

Functional foods include whole grains, phytochemical-rich fruits and vegetables, legumes, nuts, dairy products, and tea [1] that present health-promoting effects by maintaining bio-homeostasis (in mental and physical spheres) and reducing the risk of chronic diseases [2]. These foods could act by regulating central and peripheral actions, appetite, absorption, and biodefense (including immunostimulation and suppression of allergies). They can also prevent lifestyle-related diseases by reducing reactive oxygen species (ROS) production and risk of chronic diseases such as hypertension, diabetes, cancer, hypercholesterolemia, anemia, and platelet aggregation. Functional foods are expected to prevent these diseases and are used in alternative medicine [2].

The use of herbal medicinal products and supplements has increased over the past three decades; 80% of people worldwide use these products as part of primary health care [3,4]. In developed countries, herbal therapy is used with the expectation that it will promote healthier living. In developing countries, herbal medicine is an integral part of the culture of communities [4]; synthetic drugs are imported, have high costs, and thus are inaccessible to majority of the population [5].

The Cerrado, encompassing more than 204 million hectares in the central part of Brazil, is the richest tropical savanna in the world in terms of biodiversity and the second most extensive biome in South America [6]. It has been identified as one of the world's biodiversity hotspots, with around 4400 endemic plants species [7]. An estimated 30% of this biodiversity is reasonably known [8]. The Cerrado flora encompasses grasses, herbs, and 30%–40% of woody plants; usually recovered with dense indumentum, trees and bushes display contorted trunk and branches with thick and fire-resistant bark, and shiny coriaceous leaves [8]. This singular phytophysiognomy is due to natural and anthropogenic fires, long periods of drought, and characteristics of the Cerrado soil such as poverty of nutrients, aluminum abundance, and aluminum acidity [8].

Although the Cerrado flora is rich in species containing several chemical compounds with biological activities, in general, it is overlooked and its area has decreased over time. It is estimated that natural vegetation covers just 49.1% of the biome in 2000 and the losses of natural vegetation in the Cerrado is around 11,812 km^2^/year from 2005 to 2010 [9]. High land use pressure, mainly after the introduction of extensive, mechanized production of grains for exportation, is causing heavy losses of natural vegetation [10,11,12].

Plants endemic to Cerrado have been receiving increased attention as a source of bioactive compounds [13]. While the shikimate pathway is enhanced in humid forests resulting in greater production of lignified biomass, in dry forests such as the Cerrado biome, lignin precursor molecules can be replaced by polyphenols [14,15]. Therefore, phenolic compounds are very common in Cerrado plants, probably because of the exposure to water stress, high ultraviolet radiation, herbivore attacks and fungi infections [8]. Phenolic compounds, especially tannins, are directly responsible for the therapeutic activity of plants in Cerrado biome. Phenolic compounds and their potential therapeutic activity, such as anti-inflammatory and antimicrobial actions, makes Cerrado plants good candidates for bioprospecting efforts [14]. Since these compounds are found in high concentrations in many fruits and vegetables, resulting in a continuous and long-term intake of such plant phenols, phenolic compounds play a particularly important role in human health. These compounds also present antioxidant, chemoprevention, cytoprotection, anti-mutagenic, anti-estrogenic and anti-angiogenic activities [16].

The Cerrado native fruits have been used by indigenous people and played a key role in feeding the explorers and settlers of the central Brazil region [17]. Some Cerrado fruits are gaining acceptance, being used as juices, sweets, ice cream, and candies in Brazil. Studies have shown that the bioactive substances present in these fruits can act alone or together on various pathophysiologic targets to alleviate the symptoms of chronic diseases [18]. Phenolic compounds—including flavonoids, tannins, anthocyanins, and simple phenolic compounds—represent the main bioactive class of compounds that can be found in Cerrado fruits [8]. Fruits and vegetables are the main dietary sources of flavonoids, and their potential health benefits are associated with the contribution to redox regulation in cells [19]. Beside these, furanocoumarins, terpenes, stilbene derivatives, phytosterols, and fatty acids, among other kind of molecules, can be identified in this biome. Perhaps because alkaloids are commonly extracted from roots, stem bark, leaves, and wood [20], few reports about alkaloid presence in Cerrado fruits are available.

The importance of Cerrado fruits was highlighted in the work of Siqueira and colleagues [21]. A comparison of 12 Cerrado fruits with *Malus domestica* (the Red Delicious apple) revealed that nine of them—araticum (*Annona crassiflora* Mart), cagaita (*Eugenia dysenterica* DC.), cajuzinho (*Anacardium humile* St. Hil.), ingá (*Inga laurina* Willd.), jenipapo (*Genipa americana* L.), jurubeba (*Solanum paniculatum* L.), lobeira (*Solanum grandiflorum* Ruiz & Pav.), mangaba (*Hancornia speciosa* Gomes), and tucum (*Bactris setosa* Mart)—showed high levels of phenolic contents [21]. Araticum and tucum are rich in flavonoids. The fruits cajuzinho, jatobá, jurubeba, and tucum showed high content of anthocyanins. Cagaita, cajuzinho, lobeira, mangaba, and tucum showed high levels of vitamin C. The high content of bioactive compounds found in araticum, cagaita, cajuzinho, jurubeba, lobeira, magaba, and tucum corroborate the high antioxidant activity of these fruits [21]. These results indicate that a daily consumption of Cerrado fruits could protect human tissues against oxidative stress, and thus potentially prevent chronic diseases and premature aging [21].

**Figure 1:**
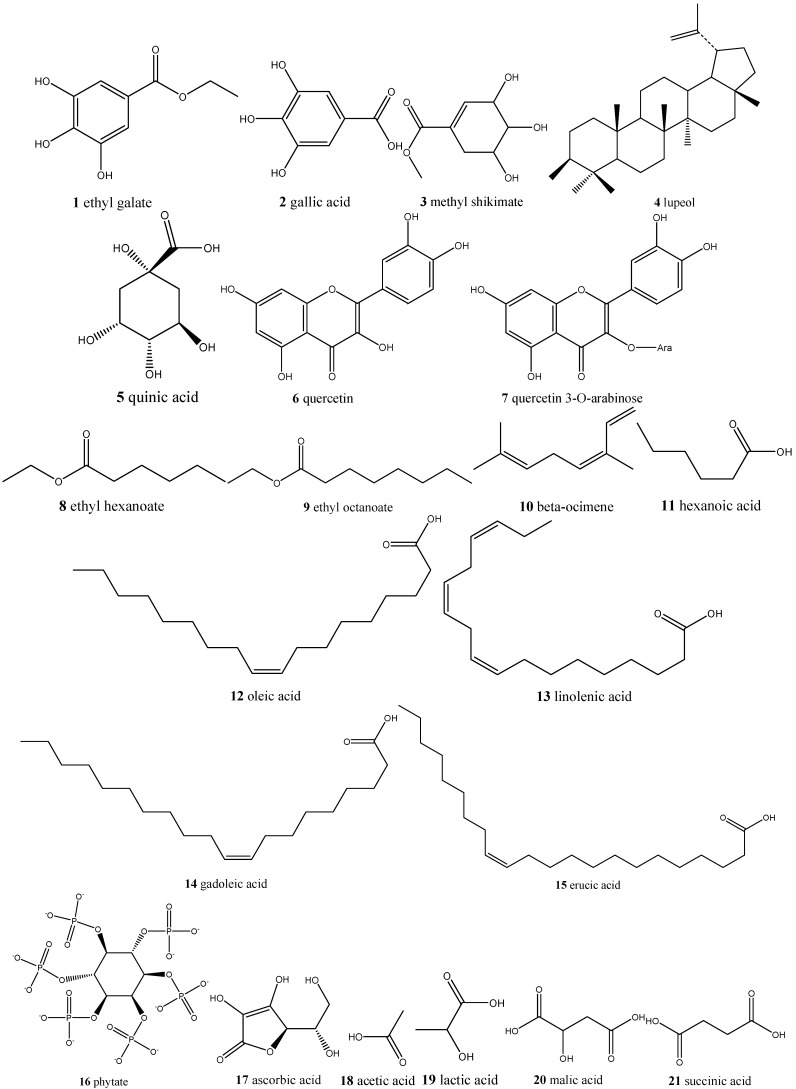

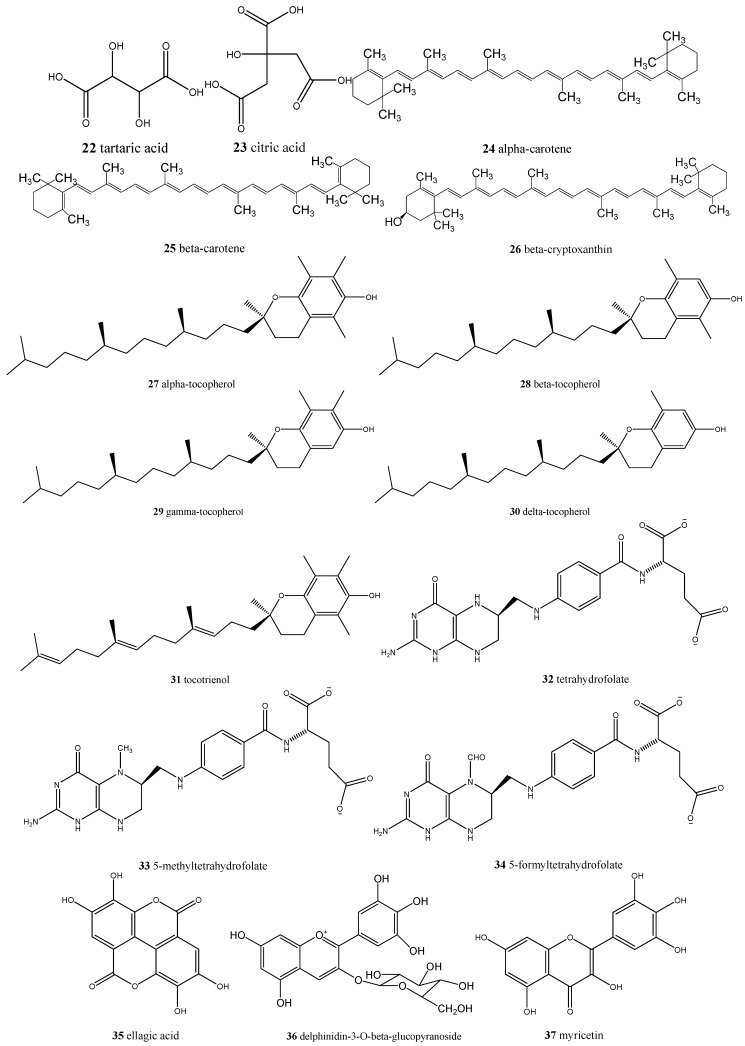

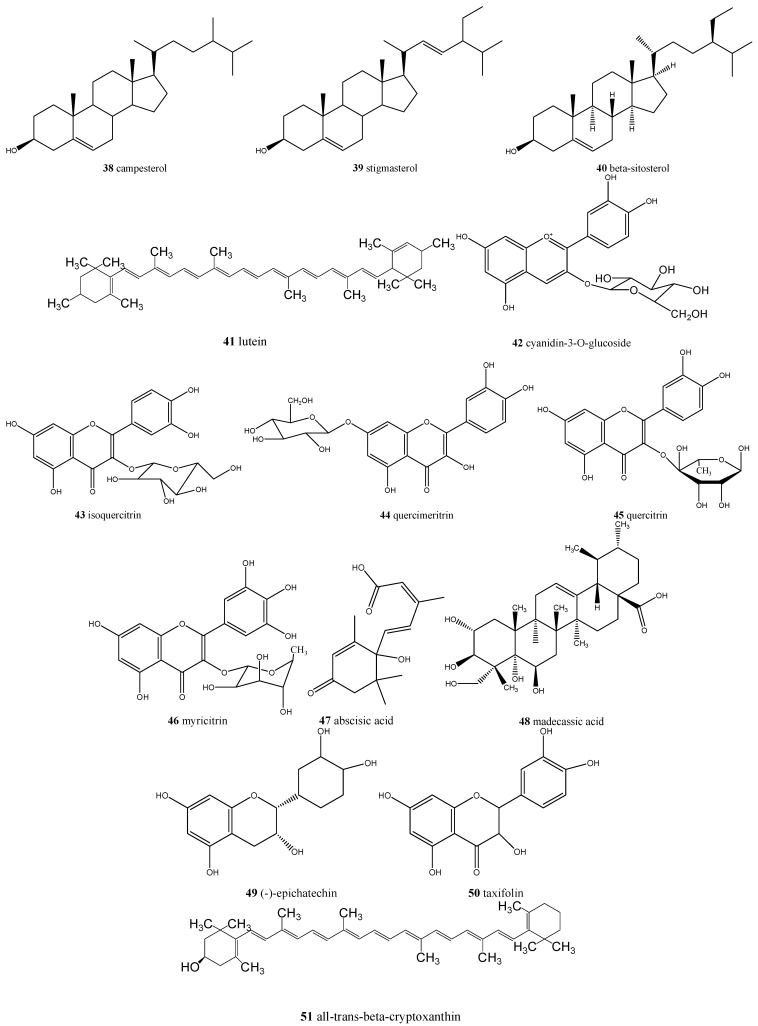

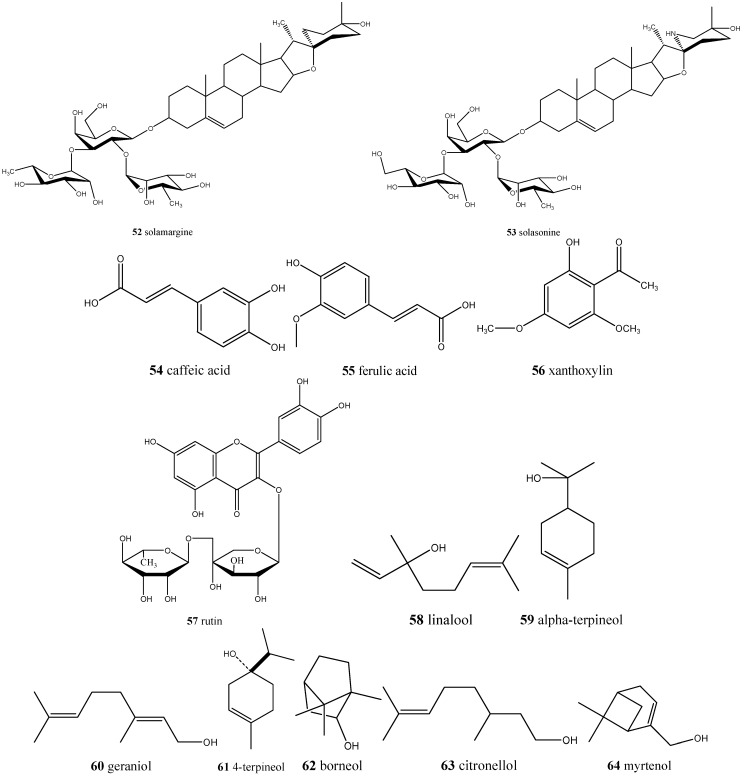
Molecular structures (**1** to **64**) of the compounds found in Cerrado fruits.

In this way, Cerrado fruits can provide a source of bioactive compounds with nutritional and functional properties beneficial to health, which should stimulate the pharmaceutical and food industries to develop new products. This would value Cerrado constituents, promoting the sustainable development of Cerrado regions and contributing to the conservation of the biodiversity of this biome [21,22]. The possibility of introducing Cerrado fruits in the form of wines, juices, pulp, or residue powdered (capsulated or bulk), could increase their use in dietary or cosmetic products and lead to trade in international markets [23]. However, more detailed agronomic, phytochemical, and pharmacologic information is needed before these advances will be feasible [18]. To compile information about Cerrado fruits and highlight the nutraceutical and pharmacologic potential of this biome, we reviewed the available literature as shown in Table 1. The functional compounds, their chemical structures (Figure 1), and their biological activities beneficial in human health are emphasized.

**Table 1:** Cerrado fruits with main metabolites and functional properties described.

Name (Scientific/Popular)	Main Metabolites ^a^	Functional Properties
**Anacardiaceae**		
*Spondias mombin*/cajá or taperebá	β-Cryptoxanthin (**26**)	Antioxidant
**Annonaceae**		
*Annona crassiflora*/araticum	Ascorbic acid (**17**), caffeic acid (**54**), quinic acid (**5**), ferulic acid (**55**), xanthoxylin (**56**), and rutin (**57**)	Antioxidant
**Arecaceae (Palmae)**		
*Mauritia flexuosa*/buriti	β-Carotene (**25**), α-carotene (**24**), lutein (**41**), and gallic acid (**2**)	Reverse clinical xerophthalmia and restore liver reserves of vitamin A
**Caryocaraceae**		
*Caryocar* spp./pequi	Ethyl galate (**1**), gallic acid (**2**), methyl shikimate (**3**), lupeol (**4**), quinic acid (**5**), quercetin (**6**), and quercetin 3-*O*-arabinose (**7**), ethyl hexanoate (**8**), ethyl octanoate (**9**), β-ocimene (**10**), and hexanoic acid (**11**)	Antioxidant, antiaging, antiproliferative, and immunomodulatory
**Leguminosae**		
*Dipteryx alata*/baru	Oleic, linoleic (**12**), linolenic (**13**), gadoleic (**14**), and erucic (**15**), phytic acid (**16**)	Antioxidant and cardiovascular diseases protection
**Myrtaceae**		
*Eugenia dysenterica*/cagaita	Ascorbic acid (**17**), acetic acid (**18**), lactic acid (**19**), malic acid (**20**), succinic acid (**21**), tartaric acid (**22**), citric acid (**23**), α-carotene (**24**), β-carotene (**25**), β-cryptoxanthin (**26**) and lycopene, α- (**27**), β- (**28**), γ- (**29**) and δ-tocopherol (**30**), tocotrienol (**31**), tetrahydrofolate (**32**), 5-methyltetrahydrofolate (**33**), 5-formyltetrahydrofolate (**34**), and ellagic acid (**35**)	Laxative and anti-obesity
*Eugenia uniflora*/pitanga	Delphinidin-3-*O*-β-glucopyranoside (**36**), myricetin (**37**), cyanidin (**38**), quercetin (**6**), ellagic acid (**35**), and proanthocyanidins	Promising natural ingredient for food and nutraceutical manufacturers
*Myrciaria cauliflora*/jabuticaba	Cyanidin-3-*O*-glucoside (**42**), delphinidin-3-*O*-glucoside (**36**), gallic acid (**2**), ellagic acid (**35**), isoquercitrin (**43**), quercimeritrin (**44**), quercitrin (**45**), myricitrin (**46**), and quercetin (**6**)	Antioxidant, anti-inflammatory, anti-diabetic, anti-obesity, could be used in chronic obstructive pulmonary disease (COPD) treatment
*Psidiumguajava*/goiaba	Ascorbic acid (**17**), myricetin (**37**), abscisic acid (**47**), and madecassic acid (**48**)	Antioxidant, antidiarrheal, antimicrobial, could reduce blood pressure and sugar, triglycerides and cholesterol blood levels, analgesic, and anti-inflammatory
*Psidium* spp./araçá	(−)-Epicatechin (**49**), gallic acid (**2**), taxifolin (**50**), quercetin (**6**), ellagic acid (**35**), all-*trans*-β-cryptoxanthin (**51**), β-carotene (**25**), and lutein (**41**)	Antimicrobial, antiproliferative, and could be involved in vasodilation
**Rubiaceae**		
*Genipa americana*/jenipapo	Campesterol (**38**), stigmasterol (**39**), and β-sitosterol (**40**)	Anti-obesity, antioxidant, and antiproliferative
**Sapotaceae**		
*Hancornia speciosa*/mangaba	β-Carotene (**25**), ascorbic acid (**17**), tocotrienol (**31**) and (6*S*)-5-formyl-5,6,7,8-tetrahydrofolate (5-FTHF) (**34**)	Antioxidant, antidiabetic, and anti-obesity
**Solanaceae**		
*Solanum lycocarpum/*lobeira	Solamargine (**52**) and solasonine (**53**)	Antidiabetic, anti-inflammatory, and anticancer

^a^ The numbers in parentheses indicate the correspondence of the molecules in Figure 1.

## 2. *Caryocar brasiliense* Camb.

*Caryocar brasiliense* Camb., known in Brazil as pequizeiro, presents commercial importance because of its edible fruit, called pequi. This fruit is characterized by the presence of several nutrients mainly in mesocarp (light-yellow, pulpy, rich in oil, vitamins and proteins) [24]. Phenolic compounds and phytosterols were detected in epicarp and external mesocarp of the pequi fruits. According to Ascari and coworkers, ethyl galate (**1**), gallic acid (**2**), methyl shikimate (**3**), and lupeol (**4**) were detected in pequi pulp, using nuclear magnetic resonance (NMR) [22]. On the other hand, using electrospray ionization mass spectrometry (ESI-MS), Roesler and coworkers detected potent antioxidants in pequi pulp, such as gallic acid (**2**), quinic acid (**5**), quercetin (**6**), and quercetin 3-*O*-arabinose (**7**) [25].

The pequi fruit has a characteristic flavor, making it a valued spice in the regional cuisine [26]. The flavor of some Cerrado fruits is due to the combination of the volatile molecules present in the essential oils of these fruits. Hydrocarbons, fatty acids, and terpenoids have been identified in the pequi fruit essential oil; esters are the predominant molecule class in this fraction [26]. The esters ethyl hexanoate (**8**) and ethyl octanoate (**9**) and the acyclic monoterpene β-ocimene (**10**) are the major components of the pequi fruit essential oil [26,27,28]. Ethyl hexanoate (**8**) is a colorless liquid with the intense fruity odor characteristic of many fruits [29], including pineapples [30], green apples, and strawberries [31] and of beverages such as wine [31], beer [32], Chinese liqueurs, and Japanese sakes [33]. Ethyl octanoate (**9**) is a liquid with a sweet, fruity-floral odor that is present in the essential oils of many fruits and in the alcoholic beverages fermented from them [31]. β-Ocimene (**10**) can be found in the essential oil of many fruits, but its use is generally restricted to perfumery [34]. The combination of these three substances may produce the sweet and floral taste characteristic of the pequi fruit [26]. Hexanoic acid (**11**) has also been identified in the essential oil of pequi fruit [26]. Although linear saturated fatty acids, when isolated, appear to contribute little to the food flavor, they are commonly used to artificially enhance flavor and aroma of foods. For instance, hexanoic acid (**11**) possesses the ability to enhance both fruity and cheesy flavors and aromas [34]. Therefore, the presence of this substance could give a slightly cheesy flavor in addition to the characteristic fruity flavor of pequi pulp [26].

Pequi carotenoid-rich oil is efficient in reducing tissue injuries in runners, particularly in women, and in reducing DNA damage in both genders, making this oil a good candidate for use as an antioxidant and an antiaging supplement [35,36,37,38]. Because of its antioxidant potential, the pequi oil has potential against tumor growth, to increase lymphocyte-dependent immunity, and reduce the adverse effects associated with doxorubicin-induced oxidative damage to normal cells [39]. Besides the oil, the pulp also has highly efficient antioxidant activity, perhaps because of the potent natural antioxidants detected in the fruit [25], as described above.

## 3. *Dipteryx alata* Vog.

The baru nut is a seed of the Baruzeiro plant (*Dipteryx alata* Vog.), a species of shrub belonging to the Fabaceae family, which is native to the Cerrado. This species produces fruit from July to October [40]. Despite the extreme climatic conditions of the Cerrado biome, baru nuts contain high-quality proteins and lipids, mainly unsaturated fatty acids such as oleic (**12**), linolenic (**13**), gadoleic (**14**) and erucic (**15**) acids [41,42]. The baru fruit also contains calcium, iron, and zinc, as well as phytate and tannins. Tannins and others polyphenols are important to human health because of their antioxidant properties [43,44].

The consumption of aqueous and ethyl acetate extracts of the baru nut by rats supplemented orally with iron provided tissue protection against iron-induced oxidative stress. This activity might be attributed to the phytic acid (**16**), but it is possible that phenolic compounds may also be involved [45]. Dietary supplementation of mildly hypercholesterolemic subjects with baru nuts improves serum lipid parameters; this fruit might therefore be included in diets aiming at reducing cardiovascular diseases. Baru almonds reduced total cholesterol, low-density lipoprotein cholesterol, and non–high-density lipoprotein cholesterol in subjects consuming a diet that included this part of fruit (20 g/day for 12 weeks) [44].

## 4. *Eugenia* spp.

The species of *Eugenia* genus belongs to the Myrtaceae family. Among the species of native edible fruit from the Brazilian Cerrado, “cagaita” (*Eugenia dysenterica* DC.) is popular with the local population. Its fruits are tasty and rich in nutritional substances, such as ascorbic acid (**17**) (vitamin C), proteins, lipids and dietary fiber [46]. According to Schwan and coworkers [47] the pulp of *E. dysenterica* fruits contains organic acids (acetic acid (**18**), lactic acid (**19**), malic acid (**20**), succinic acid (**21**), tartaric acid (**22**), and citric acid (**23**)) and carbohydrates (mainly glucose, sucrose, and fructose). Tannins also represent a phenolic class present in cagaita fruits [48]. The work of Cardoso and colleagues [49], using high performance liquid chromatography (HPLC) with a diode array detector and HPLC with fluorescence detection, showed that cagaita fruits contain other substances, such as carotenoids (α-carotene (**24**), β-carotene (**25**), β-cryptoxanthin (**26**), and lycopene), vitamin E (α- (**27**), β- (**28**), γ- (**29**), and δ-tocopherol (**30**) and tocotrienol (**31**)) and folates (tetrahydrofolate (**32**), 5-methyltetrahydrofolate (**33**), and 5-formyltetrahydrofolate (**34**)). Ellagic acid (**35**) was also found in cagaita commercial pulp [50].

The consumption of this fruit provides a portion of the recommended daily amounts of vitamin A and folates needed by children, adults, and pregnant women, contributing to the importance of this food [49]. This fruit also has laxative properties, according to popular use [51]. Lima and colleagues detected a 7 kDa peptide using a semipreparative HPLC column, MALDI-TOF, and Tris/Tricine SDS-PAGE of HPLC fractions [52]. This molecule showed laxative properties in experiments carried out in rats after charcoal meal administrations, increasing intestinal motility by 15% [52]. A recent study demonstrated that phenolic-rich extracts from cagaita fruits affect obesity and metabolism problems caused by intake of a high-fat and high-sucrose diet in male mice. The increase in body weight was attenuated by the administration of the cagaita extracts to the animals [53].

Pitanga fruits (*Eugenia uniflora* L.) can be found in various biomes, such as the Brazilian Cerrado, and are rich in phenolic substances (hydroxybenzoic acids, hydroxycinnamic acids, and flavonoids) and anthocyanin. Delphinidin-3-*O*-β-glucopyranoside (**36**) was detected in pitanga fruits using HPLC, NMR, and liquid chromatography-mass spectrometry (LC-MS) analyses [53]. Myricetin (**37**), cyanidin (**38**), quercetin (**6**), ellagic acid (**35**), and proanthocyanidins were found in pitanga residue powder, consisting of residual fruit pulps, seeds, and peels after juice extraction. It makes pitanga-dried residue a promising natural ingredient for food and nutraceutical manufacturers, due to their rich bioactive compound content [54]. In this same genus, the species *Eugenia calycina* Cambess is commonly referred to as “red pitanga or pitanga cherry of Cerrado”, a fruit widely consumed in this area of Brazil [55]. However, there are few reports regarding *E. calycina* phytochemical profile and functional properties.

## 5. *Genipa americana* L.

*Genipa americana* L., belonging to the Rubiaceae family, is a species widely distributed in tropical Central and South America, including the Cerrado biome. It yields an edible fruit popularly known as genipap or jenipapo [56]. Costa and colleagues have reported that the pulp of this fruit and seeds possess phytosterols such as campesterol (**38**), stigmasterol (**39**), and β-sitosterol (**40**) [57]. Phytosterols are present in high levels in genipap nuts and pulps, and this concentration can be compared with those in kidney bean, soybean, pecan, cashew nut, peanut, peanut oil, olive oil, and soybean [58]. Phytosterols have the potential to decrease the levels of serum low-density lipoprotein (LDL) cholesterol, which make them useful to the development of some foods enriched with these plant sterols [59,60].

Omena and colleagues evaluated the antioxidant activities of genipap pulp using the TBARS inhibition assay, which indicates protection against lipid peroxidation [61]. Ethanolic extracts of the pulp provide an acetylcholinesterase inhibition zone similar to that of the positive control, carbachol, suggesting that this extract is a potential antioxidant supplement for use in the human diet and in the pharmaceutical and cosmetic industries [61]. Conceição and coauthors [62] verified the effect of *G. americana* fruit ethanolic extract on the proliferation and differentiation of trophoblast-like cells; results showed that the extract did not cause cytotoxicity or any interference in cell differentiation. However, a significant antiproliferative state related to inhibition and reactivation of the tested cells was observed. These results suggest that the ethanolic extract of *G. americana* may affect placental cell regulation [62].

## 6. *Hancornia speciosa* Gomes

*Hancornia speciosa* Gomes, known as mangabeira, yields a round-shaped fruit, known as mangaba, which has a fragile peel that is yellow-green in color with red spots. The pulp that is rich in vitamin C, vitamin E, and folate is green and viscous with numerous beige seeds [63]. Mangaba has dietary fiber content similar to fruits considered good sources of dietary fiber, including tangerine and pear [64]. Moreover, carotenoids, vitamin C, vitamin E, and folate compounds have been identified in mangaba pulp. Among the carotenoids, β-carotene (**25**) was the major component (52.6% of total carotenoids). Among vitamins, ascorbic acid (**17**) and tocotrienol (**31**) were the major vitamin C and vitamin E compounds, respectively. (6*S*)-5-formyl-5,6,7,8-tetrahydrofolate (5-FTHF) (**34**) was the prevalent folate in mangaba pulp [63].

Because of the presence of these natural antioxidants, mangaba pulp is considered to have great radical-scavenging capacity [65]. The high fiber content of mangaba could be important for human health since it may help improve the glycemic index of the diet, glycemic control, and weight control [63,66]. Moreover, the high antioxidant and folate contents of mangaba pulp could reduce the risk of development of several chronic-degenerative diseases, such as cancer and cardiovascular diseases [63,67,68].

## 7. *Mauritia flexuosa* L.f.

Buriti (*Mauritia flexuosa* L.f.) is a palm found in the Cerrado biome. It produces a fruit with a characteristic color that ranges from yellow to dark reddish brown [69]. These fruits contain high levels of provitamin A carotenoids, mainly β-carotene (**25**) [70]. In recent work, total phenolics from buriti fruits were quantified using Folin-Ciocalteau reagent. Gallic acid (**2**) was detected in amounts higher than that in other species of the same family (Arecaceae), such as *Oenocarpus bacaba* Mart. (bacaba), *Euterpe edulis* (jussara), *Euterpe oleracea* (açaí), and *Copernicia prunifera* (carnaúba) [71]. These authors also determined the chromatographic profile of carotenoids, identifying α-carotene (**24**), β-carotene (**25**), and lutein (**41**) (a xanthophyll with two hydroxyl groups). These results confirmed the predominance of β-carotene (**25**), an important vitamin A precursor. Tocopherol (**27**–**30**) is also found in buriti fruits. However, the content of vitamin E in fruits and vegetables can be affected by species, maturity, growing conditions (weather, growing season, intensity of sunlight, and soil), and time and manner of harvesting. After harvesting, the concentration of vitamin E can also decline based on factors such as processing procedures, storage time and conditions, sample preparation, and analytical method variation [72].

Because of its high β-carotene (**25**) content, buriti pulp can reverse clinical xerophthalmia and restore liver reserves of vitamin A, suggesting potential use in intervention programs to combat vitamin A deficiency in countries where the fruit is available or has the potential for cultivation [73].

## 8. *Myrciaria cauliflora* (DC) Berg

*Myrciaria cauliflora* (DC) Berg, popularly known as jabuticabeira or “Brazilian grape tree,” is native to Brazil and provides a typical fruit known as jabuticaba that is widely consumed mainly in the southeastern part of this country [74,75]. This fruit has been called a “super fruit,” mainly in the food industry. Its protective effects can be attributed, in part, to phenolic secondary metabolites [76]. The jabuticaba peel is a good source of nutrients such as minerals, soluble and insoluble fibers, and a group of phenolic compounds known as anthocyanins [77,78]. The major anthocyanins found in jabuticaba peels are cyanidin-3-*O*-glucoside (**42**) and delphinidin-3-*O*-glucoside (**36**). The presence of these substances accounts for the fruit’s antioxidant activity [79]. Other phenolic compounds were detected in jabuticaba, including gallic acid (**2**), ellagic acid (**35**), isoquercitrin (**43**), quercimeritrin (**44**), quercitrin (**45**), myricitrin (**46**), and quercetin (**6**) [75].

Several epidemiologic works show that diets rich in dark-colored fruits reduce the incidence of cardiovascular diseases, diabetes, cancer, and stroke [80]. The presence of depsides and anthocyanins, which have strong antioxidant, anti-inflammatory, anti-diabetic, and anti-obesity properties, may explain this effect. Moreover, these compounds are potential treatments for chronic obstructive pulmonary disease [75,81,82,83,84].

## 9. *Psidium* spp.

The *Psidium* genus, native to tropical and subtropical America, includes around 100 species of trees and shrubs. *Psidium* *guajava* L., known as goiabeira, is the most economically important species [17,85]. Its fruits contain many vitamins and minerals and have large amounts of phenolic substances. Guava fruits contain four times more ascorbic acid (**17**) than orange and also contain flavonoids, triterpenoids, and other biologically active secondary compounds [86]. The guava pulp contains total phenols and total flavonoids in amounts higher than in fruits such as cajá, mango, pineapple, and tamarind [87]. In a study of seven cultivars of guava fruits, the flavonoid myricetin (**37**), sesquiterpenoid abscisic acid (**47**), and triterpene madecassic acid (**48**) were detected in all varieties by mass spectrometry [88].

The extracts of the *P. guajava* cultivars are potent free-radical scavengers and may be used as a good source of natural antioxidants for food, pharmaceutical, medical, and commercial uses [88]. Although the ripe fruit could be a laxative, the unripe fruit is used as astringent and antidiarrheal agent in popular medicine [89]. On the other hand, a galactose-specific lectin, isolated from ripe fruit, was shown to bind to *Escherichia coli* (a common diarrhea-causing bacterium), preventing its adhesion to the intestinal wall and thus preventing diarrhea [90]. Previous reviews pointed that the fruit or its juice have antimicrobial activity and could reduce blood pressure and serum glucose, triglyceride, and cholesterol levels, and also ameliorate rheumatism (analgesic and anti-inflammatory effects) [91].

*Psidium cattleianum* Sabine, *P. firmum* O. Berg, and *P. guineensis* Sw., known as araçazeiro, present yellow or red berries with potential commercial interest [17,92]. The fruit, araçá, presents a high content of dietary fiber, folate, and vitamin C, around three or four times higher than other citric fruits, and a low lipid content, indicating that this fruit is nutritionally valuable [93,94]. Araçá has a total phenolic content higher than strawberry (*Fragaria ananassa* Duch.) and grape (*Vitis vinifera* L.) [92]. HPLC-UV has shown that (−)-epicatechin (**49**) and gallic acid (**2**) are the major phenolic compounds present in araçá [92]. Mass spectrometry (MS) has identified gallic acid (**2**) and taxifolin (**50**) as the major phenolic compounds present in araçá [95]. Quercetin (**6**) and ellagic acid (**35**) were also detected in araçá [50]. Even though araçá is not a good source of carotenoids, these compounds were identified in araçá pulp using MS. The all-*trans*-β-cryptoxanthin (**51**) was the major carotenoid, representing 34% of the total carotenoid content in this fruit, followed by β-carotene (**25**) and lutein (**41**), corresponding to 26% and 20% of the total content, respectively [95].

Through vasodilation and reduction in blood pressure, (−)-epicatechin (**49**) could contribute to reducing risks of cardiovascular diseases [96]. Moreover, polyphenols could play an important role in cancer prevention through epigenetic mechanisms, mainly by DNA methylation, histone modification, or regulation of mRNA expression [97]. Araçá extracts present antimicrobial effects against *Salmonella enteritidis*, possibly owing to the presence of phenolic compounds, which could destabilize the bacterial cell membrane responsible for prokaryotic respiration [92]. Moreover, araçá extracts reduced survival rates of breast and colon cancer cells (MCF-7 and Caco-2, respectively) *in vitro*, by mechanisms not yet described, indicating an antiproliferative effect of araçá extracts [92].

## 10. *Solanum lycocarpum* St. Hill

*Solanum lycocarpum* St. Hill (Solanaceae) is a native shrub that produces a fruit popularly known as lobeira. Glycoalkaloids and polyphenols are the most common elements obtained from *S. lycocarpum*, which has a great importance for use in food and as medicine in Cerrado [98]. This fruit contains two major glycoalkaloids, solamargine (**52**) and solasonine (**53**) [99]. These compounds are structurally similar, with the same steroidal moiety, solasodine, differing only in their sugar chain moieties, solatriose for solasonine and chacotriose for solamargine [100].

Several alkaloids isolated from natural herbs exhibit antiproliferative, antibacterial, antiviral, insecticidal, and antimetastatic effects on various types of cancers both *in vitro* and *in vivo* [101]. The glycoalkaloids solamargine (**52**) and solasonine (**53**) were identified in various species of the Solanaceae family [102], but *S. lycocarpum* stands out for the production of these compounds [103]. Solamargine (**52**) and solasonine (**53**) could function as antidiabetic compounds, since they inhibit the increase of rat serum glucose levels, probably by suppressing the transfer of sucrose from the stomach to the small intestine [100]. Because of the presence of these glycoalkaloids, high-fiber *S. lycocarpum* flour reduced plasma glucose in diabetic rats. In *S. lycocarpum* flour-treated diabetic rats (TDRs), serum glucose, water and food intake, urine excretion, and urine sodium concentration reduced compared with diabetic control rats (DCRs). In addition, TDRs did not show signs of kidney hypertrophy, unlike those in the DCR group. These results suggest that the use of *S. lycocarpum* flour can be an effective support in diabetes mellitus treatment [104]. Moreover, an alkaloid fraction of *S. lycocarpum* fruits induced a dose-dependent reduction in edema formation and leukocyte migration [105], suggesting that *S. lycocarpum* solamargine (**52**) and solasonine (**53**) may act as anti-inflammatory compounds.

Solamargine (**52**) and solasonine (**53**) have antitrypanosomal [98,106], schistosomicidal [107], antiherpetic [108], antifungal [109,110], immunomodulatory [99], and anticancer [111,112,113,114] activities. Formulations based on *Solanum*-derived glycoalkaloids may be useful for topical therapy of several skin disorders, mainly skin cancer, but also leishmaniasis, herpes, and dermatophytosis [103,115]. Recently, investigators reported a promising topical formulation containing solamargine (**52**) and solasonine (**53**) extracted from *S. lycocarpum* that allows the penetration of the glycoalkaloids into deeper skin layers, where cancerous lesions commonly take place [103].

## 11. *Spondias mombin* L.

*Spondias mombin* L. is a member of the Anacardiaceae family and is found in the tropical areas of America, Asia, and Africa. The fruit of this tree, known as cajá or taperebá, is a small ovoid drupe with thin yellow skin and a sour-sweet taste [116]. The cajá pulp has high levels of micronutrients, such as potassium, magnesium, phosphorus, and copper. Moreover, high levels of phenolic and carotenoids compounds are present in this fruit, resulting in a high level of cajá antioxidant activity above the fruits average [117]. Five different carotenoids were identified in cajá pulp, with β-cryptoxanthin (**26**) the major compound (48% of the total carotenoid amount) [117]. Its presence gives cajá high nutritional and functional value that may help prevent various diseases, including cardiovascular disorders [117,118].

## 12. Other Cerrado Fruits

*Annona crassiflora* Mart. is a tree 4–8 m high; it produces an oval-round fruit known as araticum [17]. Its yellow pulp is sweet, with a strong, peculiar aroma [119]. Important bioactive molecules have been identified using ESI-MS in araticum fruit, including ascorbic acid (**17**), caffeic acid (**54**), quinic acid (**5**), ferulic acid (**55**), xanthoxylin (**56**) and rutin (**57**) [120]. These compounds could be involved with the antioxidant activity observed in this fruit [119,121].

The pulp and kernel of three palm fruits distributed in Brazilian Cerrado, *Syagrus oleracea* (guariroba), *Syagrus romanzoffiana* (jerivá), and *Acrocomia aculeata* (macaúba), have been studied [122]. Jerivá pulp had the highest percentage of fibers, followed by guariroba and macaúba. Pulp oils were richer in the bioactive compounds carotenoids and tocopherols than kernel oils. Jerivá pulp oil showed considerable carotenoid and tocopherol amounts, especially α-tocopherol (**27**), thus representing an important source of antioxidant vitamins A and E [122].

Some Cerrado fruits have potential use in production of wines, such as *Campomanesia pubescens* (DC.) O. Berg (gabiroba) and *Myrciaria jaboticaba* Berg (jabuticaba). Gabiroba is a Cerrado native fruit consumed fresh or used in the production of homemade ice cream, jam, and juices [17]. Jabuticaba and gabiroba wine chemical characterization was performed [123]. In both wines, the following monoterpenic alcohols were detected: linalool (**58**), α-terpineol (**59**), and geraniol (**60**). In contrast, 4-terpineol (**61**), borneol (**62**), citronellol (**63**), and myrtenol (**64**) were detected only in gabiroba wine. The monoterpenic compounds play an important role in the varietal flavor of wines [124].

## 13. Conclusions

Brazilian Cerrado is an important source of food or medicine to the local communities. The fruits found in this biome contain several nutrients, such as fibers, micronutrients, and vitamins A, C, and E. Moreover, secondary metabolites with biological activities were identified in Cerrado fruits, especially phenolic compounds such as tannins, flavonoids, anthocyanins, and single phenols. Because of their rich bioactive compound content, Cerrado fruits could be promising ingredients for nutraceutical and pharmaceutical manufacturers, expanding the market utility of these fruits. The information presented in this review should be useful for further exploitation of several species found in the Cerrado biome.

## References

[1] Mirmiran P., Bahadoran Z., Azizi F. (2014). Functional foods-based diet as a novel dietary approach for management of type 2 diabetes and its complications: A review. World J. Diabetes.

[2] Mitsuoka T. (2014). Development of functional foods. Biosci. Microbiota Food Health.

[3] WHO, IUCN, WWF (1993). Guidelines on the Conservation of Medicinal Plants.

[4] Ekor M. (2014). The growing use of herbal medicines: Issues relating to adverse reactions and challenges in monitoring safety. Front. Pharmacol..

[5] Silva V.A., Freitas A.F.R., Pereira M.S.V., Oliveira C.R.M., Diniz M.F.F.M., Pessôa H.L.F. (2011). Eficácia antifúngica dos extratos da *Lippia sidoides* Cham. e *Matricaria recutita* Linn. sobre leveduras do Gênero *Candida*. BioFar.

[6] Sano E.E., Rosa R., Brito J.L., Ferreira L.G. (2010). Land cover mapping of the tropical savanna region in Brazil. Environ. Monit. Assess..

[7] Myers N., Mittermeier R.A., Mittermeier C.G., da Fonseca G.A., Kent J. (2000). Biodiversity hotspots for conservation priorities. Nature.

[8] Fank-de-Carvalho S.M., Somavilla N.S., Marchioretto M.S., Báo S.N., Lo Y., Blanco J.A., Roy S. (2015). Plant structure in the Brazilian neotropical savannah species. Biodiversity in Ecosystems—Linking Structure and Function.

[9] Beuchle R., Grecchi R.C., Shimabukuro Y.E., Seliger R., Eva H.D., Sano E., Achard F. (2015). Land cover changes in the Brazilian Cerrado and Caatinga biomes from 1990 to 2010 based on a systematic remote sensing sampling approach. Appl. Geogr..

[10] Jepson W. (2005). A disappearing biome? Reconsidering land cover change in the Brazilian savanna. Geogr. J..

[11] Klink C.A., Machado R.B. (2005). Conservation of the Brazilian Cerrado. Conserv. Biol..

[12] Silva J.F., Farinas M.R., Felfili J.M., Klink C.A. (2006). Spatial heterogeneity, land use and conservation in the Cerrado region of Brazil. J. Biogeogr..

[13] Violante I.M., Hamerski L., Garcez W.S., Batista A.L., Chang M.R., Pott V.J., Garcez F.R. (2012). Antimicrobial activity of some medicinal plants from the Cerrado of the centralwestern region of Brazil. Braz. J. Microbiol..

[14] Albuquerque U.P., Ramos M.A., Melo J.G. (2012). New strategies for drug discovery in tropical forests based on ethnobotanical and chemical ecological studies. J. Ethnopharmacol..

[15] Gottlieb O.R., Kaplan M.A.C., Borin M.R.M. (1996). Biodiversidade—Um Enfoque Químico-Biológico.

[16] Sun Q., Heilmann J., Konig B. (2015). Natural phenolic metabolites with anti-angiogenic properties—A review from the chemical point of view. Beilstein J. Org. Chem..

[17] Vieira R.F., Costa T.S.A., Silva D.B., Ferreira F.R., Sano S.M. (2006). Frutas Nativas da Região Centro-Oste do Brasil.

[18] Oliveira V.B., Yamada L.T., Fagg C.W., Brandão M.G.L. (2012). Native foods from Brazilian biodiversity as a source of bioactive compounds. Food Res. Int..

[19] Rice-Evans C.A., Miller N.J., Paganga G. (1996). Structure-antioxidant activity relationships of flavonoids and phenolic acids. Free Radic. Biol. Med..

[20] Tajkarimi M.M., Ibrahim S.A., Cliver D.O. (2010). Antimicrobial herb and spice compounds in food. Food Control.

[21] Siqueira E.M., Rosa F.R., Fustinoni A.M., de Sant’Ana L.P., Arruda S.F. (2013). Brazilian savanna fruits contain higher bioactive compounds content and higher antioxidant activity relative to the conventional red delicious apple. PLoS ONE.

[22] Rocha W.S., Lopes R.M., Silva D.B., Vieira R.F., Silva J.P., Agostini-Costa T.S. (2011). Total phenolics and condensed tannins in native fruits from brazilian savanna. Rev. Bras. Frutic..

[23] Georgiev V., Ananga A., Tsolova V. (2014). Recent advances and uses of grape flavonoids as nutraceuticals. Nutrients.

[24] Vieira R.F., Martins M.V.M. (2000). Recursos genéticos de plantas medicinais de cerrado: Uma compilação de dados. Braz. J. Med. Plants.

[25] Roesler R., Catharino R.R., Malta L.G., Eberlin M.N., Pastore G. (2008). Antioxidant activity of *Caryocar brasiliense* (pequi) and characterization of components by electrospray ionization mass spectrometry. Food Chem..

[26] Geőcze K.C., Barbosa L.C.A., Fidêncio P.H., Silvério F.O., Lima C.F., Barbosa M.C.A., Ismail F.M.D. (2013). Essential oils from pequi fruits from the Brazilian Cerrado ecosystem. Food Res. Int..

[27] Damiani C., Vilas Boas E.V.B., Ferri P.H., Pinto D.M., Rodrigues L.J. (2009). Volatile compounds profile of fresh-cut peki fruit stored under different temperatures. Ciênc. Tecnol. Aliment..

[28] Maia J.G.S., Andrade E.H.A., Silva M.H.L. (2008). Aroma volatiles of pequi fruit (*Caryocar brasiliense* Camb.). J. Food Compos. Anal..

[29] Dury-Brun C., Hirata Y., Guillard V., Ducruet V., Chalier P., Voilley A. (2008). Ethyl hexanoate transfer in paper and plastic food packaging by sorption and permeation experiments. J. Food Eng..

[30] Elsss S., Preston C., Hertzig C., Heckel F., Richling E., Schreier P. (2005). Aroma profiles of pineapple fruit (*Ananascomosus* [L.] Merr.) and pineapple products. LWT.

[31] Sumby K.M., Grbin P.R., Jiranek V. (2010). Microbial modulation of aromatic esters in wine: Current knowledge and future prospects. Food Chem..

[32] Vanderhaegen B., Delvaux F., Daenen L., Verachtert H., Delvaux F.R. (2007). Aging characteristics of different beer types. Food Chem..

[33] Han S.Y., Pan Z.Y., Huang D.F., Ueda M., Wang X.N., Lin Y. (2009). Highly efficient synthesis of ethyl hexanoate catalyzed by CALB-displaying *Saccharomyces cerevisiae* whole-cells in non-aqueous phase. J. Mol. Catal. B Enzym..

[34] Surburg H., Panten J. (2006). Common Fragrance and Flavor Materials: Preparation, Properties and Uses.

[35] Miranda-Vilela A.L., Akimoto A.K., Alves P.C., Pereira L.C., Goncalves C.A., Klautau-Guimaraes M.N., Grisolia C.K. (2009). Dietary carotenoid-rich pequi oil reduces plasma lipid peroxidation and DNA damage in runners and evidence for an association with MnSOD genetic variant-Val9Ala. Genet. Mol. Res..

[36] Miranda-Vilela A.L., Pereira L.C., Goncalves C.A., Grisolia C.K. (2009). Pequi fruit (*Caryocar brasiliense* Camb.) pulp oil reduces exercise-induced inflammatory markers and blood pressure of male and female runners. Nutr. Res..

[37] Amaral L.F., Moriel P., Foglio M.A., Mazzola P.G. (2014). *Caryocar brasiliense* supercritical CO_2_ extract possesses antimicrobial and antioxidant properties useful for personal care products. BMC Complement. Altern. Med..

[38] Miranda-Vilela A.L., Alves P.C.Z., Akimoto A.K., Lordelo G.S., de Nazare Klautau-Guimarães M., Grisolia C.K. (2011). Under increased hydrogen peroxide conditions, the antioxidant effects of pequi oil (*Caryocar brasiliense* Camb.) to decrease DNA damage in runners are influenced by sex, age and oxidative stress-related genetic polymorphisms. Free Radic. Antioxid..

[39] Miranda-Vilela A.L., Grisolia C.K., Longo J.P., Peixoto R.C., de Almeida M.C., Barbosa L.C., Roll M.M., Portilho F.A., Estevanato L.L., Bocca A.L. (2014). Oil rich in carotenoids instead of vitamins C and E as a better option to reduce doxorubicin-induced damage to normal cells of Ehrlich tumor-bearing mice: Hematological, toxicological and histopathological evaluations. J. Nutr. Biochem..

[40] Siqueira A.C.M.F., Nogueira J.C.B., Morais E., Kageyama P.Y., Murgel J.M.T., Zandarin M.A. (1986). O cumbaru—*Dipteryx alata* Vog, Estudo de diferentes procedências e progênies. Bol. Téc. Inst. Florest..

[41] Oliveira Sousa A.G., Fernandes D.C., Alves A.M., de Freitas J.B., Naves M.M.V. (2011). Nutritional quality and protein value of exotic almonds and nut from the Brazilian savanna compared to peanut. Food Res. Int..

[42] Martins D.T.O., Lima J.C.S., Rao V.S.N. (2002). The acetone soluble fraction from bark extract of *Stryphnodendron adstringens* (Mart.) coville inhibits gastric acid secretion and experimental gastric ulceration in rats. Phytother. Res..

[43] Martins F.S., Borges L.L., Paula J.R., Conceição E.C. (2013). Impact of different extraction methods on the quality of *Dipteryx alata* extracts. Rev. Bras. Farmacogn..

[44] Bento A.P., Cominetti C., Simoes Filho A., Naves M.M. (2014). Baru almond improves lipid profile in mildly hypercholesterolemic subjects: A randomized, controlled, crossover study. Nutr. Metab. Cardiovasc. Dis..

[45] Siqueira E.M.A., Marin A.M.F., da Cunha M.S.B., Fustinoni A.M., de Sant’Ana L.P., Arruda S.F. (2012). Consumption of baru seeds (*Dipteryx alata* Vog.), a Brazilian savanna nut, prevents iron-induced oxidative stress in rats. Food Res. Int..

[46] Genovese M.I., Silva Pinto M., Gonçalves A.E.S.S., Lajolo F.M. (2008). Bioactive compounds and antioxidant capacity of exotic fruits and commercial frozen pulps from Brazil. Food Sci. Technol. Int..

[47] Schwan R.F., Mendonça A.T., Santos J.J., Rodrigues V., Wheals A.E. (2001). Microbiology and physiology of cachaça (aguardente) fermentations. Antonie Van Leeuwenhoek.

[48] Couto R.O., Araújo R.R., Tacon L.A., Conceição E.C., Bara M.T.F., Paula J.A.M., Freitas L.A.P. (2011). Development of a phytopharmaceutical intermediate product. Dry. Technol..

[49] Cardoso L.M., Martino H.S.D., Moreira A.V.B., Ribeiro S.M.R., Pinheiro-Sant’Ana H.M. (2011). Cagaita (*Eugenia dysenterica* DC.) of the Cerrado of Minas Gerais, Brazil: Physical and chemical characterization, carotenoids and vitamins. Food Res. Int..

[50] Gonçalves A.E.S.S., Lajolo F.M., Genovese M.I. (2010). Chemical composition and antioxidant/antidiabetic potential of Brazilian native fruits and commercial frozen pulps. J. Agric. Food Chem..

[51] Palhares D. (2003). Caracterizac¸ ão farmacognóstica das folhas de *Eugenia dysenterica* DC (Myrtaceae Jussieu). Rev. Lecta.

[52] Lima T.B., Silva O.N., Oliveira J.T., Vasconcelos I.M., Scalabrin F.B., Rocha T.L., Grossi-de-Sa M.F., Silva L.P., Guadagnin R.V., Quirino B.F. (2010). Identification of *E. dysenterica* laxative peptide: A novel strategy in the treatment of chronic constipation and irritable bowel syndrome. Peptides.

[53] Donado-Pestana C.M., Belchior T., Genovese M.I. (2015). Phenolic compounds from cagaita (*Eugenia dysenterica* DC.) fruit prevent body weight and fat mass gain induced by a high-fat, high-sucrose diet. Food Res. Int..

[54] Correia R.T., Borges K.C., Medeiros M.F., Genovese M.I. (2012). Bioactive compounds and phenolic-linked functionality of powdered tropical fruit residues. Food Sci. Technol. Int..

[55] Vonbulow J.F.W., Carmona R., Parente T.V. (1994). Treatment and storage of *Eugenia calycina* seeds. Pesqui. Agropecu. Bras..

[56] Ono M., Ishimatsu N., Masuoka C., Yoshimitsu H., Tsuchihashi R., Okawa M., Kinjo J., Ikeda T., Nohara T. (2007). Three new monoterpenoids from the fruit of Genipa americana. Chem. Pharm. Bull..

[57] Costa P.A., Ballus C.A., Teixeira-Filho J., Godoy H.T. (2010). Phytosterols and tocopherols content of pulps and nuts of Brazilian fruits. Food Res. Int..

[58] Abidi S.L. (2001). Chromatographic analysis of plant sterols in foods and vegetable oils. J. Chromatogr. A.

[59] Toivo J., Phillips K., Lampi A.-M., Piironen V. (2001). Determination of sterols in foods: Recovery of free, esterified, and glycosidic sterols. J. Food Compos. Anal..

[60] Lagarda M.J., García-Llatas G., Farré R. (2006). Analysis of phytosterols in foods. J. Pharm. Biomed. Anal..

[61] Omena C.M.B., Valentim I.B., Guedes G.d.S., Rabelo L.A., Mano C.M., Bechara E.J.H., Sawaya A.C.H.F., Trevisan M.T.S., da Costa J.G., Ferreira R.C.S. (2012). Antioxidant, anti-acetylcholinesterase and cytotoxic activities of ethanol extracts of peel, pulp and seeds of exotic Brazilian fruits. Food Res. Int..

[62] Conceição A.O., Rossi M.H., de Oliveira F.F., Takser L., Lafond J. (2011). *Genipa americana* (Rubiaceae) fruit extract affects mitogen-activated protein kinase cell pathways in human trophoblast-derived BeWo cells: Implications for placental development. J. Med. Food.

[63] Cardoso L.D.M., Reis B.D.L., Oliveira D.D.S., Pinheiro-Sant’Ana H.M. (2014). Mangaba (*Hancornia speciosa* Gomes) from the Brazilian Cerrado: Nutritional value, carotenoids and antioxidant vitamins. Fruits.

[64] Lima D.M. (2011). Tabela Brasileira de Composição de Alimentos-TACO.

[65] Rufino M., Fernandes F., Alves R., Debrito E. (2009). Free radical-scavenging behaviour of some North-East Brazilian fruits in a DPPH system. Food Chem..

[66] Kendall C.W.C., Esfahani A., Jenkins D.J.A. (2010). The link between dietary fibre and human health. Food Hydrocoll..

[67] Yang C.S., Suh N. (2013). Cancer prevention by different forms of tocopherols. Top. Curr. Chem..

[68] Picciano M.F., Yetley E.A., Coates P.M., McGuire M.K. (2009). Update on folate and human health. Nutr. Today.

[69] Lorenzi H., Souza H.M., Costa J.T.M., Cerqueira L.S.C., Ferreira E. (2004). Palmeiras Brasileiras e Exóticas Cultivadas.

[70] Rosso V.V., Mercadante A.Z. (2007). Identification and quantification of carotenoids, by HPLC-PDA-MS/MS, from Amazonian fruits. J. Agric. Food Chem..

[71] Candido T.L., Silva M.R., Agostini-Costa T.S. (2015). Bioactive compounds and antioxidant capacity of buriti (*Mauritia flexuosa* L.f.) from the Cerrado and Amazon biomes. Food Chem..

[72] Chun J., Lee J., Ye L., Exler J., Eitenmiller R.R. (2006). Tocopherol and tocotrienol contents of raw and processed fruits and vegetables in the United States diet. J. Food Compos. Anal..

[73] Mariath J.G., Lima M.C., Santos L.M. (1989). Vitamin A activity of buriti (*Mauritia vinifera* Mart) and its effectiveness in the treatment and prevention of xerophthalmia. Am. J. Clin. Nutr..

[74] Clerici M.T.P.S., Carvalho-Silva L.B. (2011). Nutritional bioactive compounds and technological aspects of minor fruits grown in Brazil. Food Res. Int..

[75] Wu S.B.A., Dastmalchi K., Long C.L., Kennelly E.J. (2012). Metabolite Profiling of Jaboticaba (*Myrciaria cauliflora*) and Other Dark-Colored Fruit Juices. J. Agric. Food Chem..

[76] Akter M.S., Oh S., Eun J.-B., Ahmed M. (2011). Nutritional compositions and health promoting phytochemicals of camu-camu (*Myrciaria dubia*) fruit: A review. Food Res. Int..

[77] Dugo P., Mondello L., Errante G., Zappia G., Dugo G. (2001). Identification of anthocyanins in berries by narrow-bore high-performance liquid chromatography with electrospray ionization detection. J. Agric. Food Chem..

[78] Borges L.L., Conceição E.C., Silveira D. (2014). Active compounds and medicinal properties of *Myrciaria* genus. Food Chem..

[79] Leite A.V., Malta L.G., Riccio M.F., Eberlin M.N., Pastore G.M., Marostica M.R. (2011). Antioxidant potential of rat plasma by administration of freeze-dried jaboticaba peel (*Myrciaria jaboticaba* Vell Berg). J. Agric. Food Chem..

[80] Crozier A., Jaganath I.B., Clifford M.N. (2009). Dietary phenolics: Chemistry, bioavailability and effects on health. Nat. Prod. Rep..

[81] Dastmalchi K., Flores G., Wu S.B., Ma C.H., Dabo A.J., Whalen K., Reynertson K.A., Foronjy R.F., D’Armiento J.M., Kennelly E.J. (2012). Edible *Myrciaria vexator* fruits: Bioactive phenolics for potential COPD therapy. Bioorg. Med. Chem..

[82] Prior R.L., Wilkes S., Rogers T., Khanal R.C., Wu X., Howard L.R. (2010). Purified blueberry anthocyanins and blueberry juice alter development of obesity in mice fed an obesogenic high-fat diet. J. Agric. Food Chem..

[83] Prior R.L., Wilkes S., Rogers T., Khanal R.C., Wu X., Hager T.J., Hager A., Howard L. (2010). Dietary black raspberry anthocyanins do not alter development of obesity in mice fed an obesogenic high-fat diet. J. Agric. Food Chem..

[84] Prior R.L., Wu X., Gu L., Hager T., Hager A., Wilkes S., Howard L. (2009). Purified berry anthocyanins but not whole berries normalize lipid parameters in mice fed an obesogenic high fat diet. Mol. Nutr. Food Res..

[85] Landrum L.R., Kawasaki M.L. (1997). The Genera of Myrtaceae in Brazil: An Illustrated Synoptic Treatment and Identification Keys.

[86] Hassimotto N.M., Genovese M.I., Lajolo F.M. (2005). Antioxidant activity of dietary fruits, vegetables, and commercial frozen fruit pulps. J. Agric. Food Chem..

[87] Paz M., Gullon P., Barroso M.F., Carvalho A.P., Domingues V.F., Gomes A.M., Becker H., Longhinotti E., Delerue-Matos C. (2015). Brazilian fruit pulps as functional foods and additives: Evaluation of bioactive compounds. Food Chem..

[88] Flores G., Wu S.B., Negrin A., Kennelly E.J. (2015). Chemical composition and antioxidant activity of seven cultivars of guava (*Psidium guajava*) fruits. Food Chem..

[89] Sanda K.A., Grema H.A., Geidam Y.A., Bukar-Kolo Y.M. (2011). Pharmacological aspects of *Psidium guajava*: An update. Int. J. Pharmacol..

[90] Coutiño R.R., Hernández C.P., Giles R.H. (2001). Lectins in fruits having gastrointestinal activity: Their participation in the hemagglutinating property of *Escherichia coli* O157:H7. Arch. Med. Res..

[91] Gutiérrez R.M., Mitchell S., Solis R.V. (2008). *Psidium guajava*: A review of its traditional uses, phytochemistry and pharmacology. J. Ethnopharmacol..

[92] Medina A.L., Haas L.I.R., Chaves F.C., Salvador M., Zambiazi R.C., da Silva W.P., Nora L., Rombaldi C.V. (2011). Araçá (*Psidium cattleianum* Sabine) fruit extracts with antioxidant and antimicrobial activities and antiproliferative effect on human cancer cells. Food Chem..

[93] Hamacek F.R., Santos P.R.G., Cardoso L.M., Ribeiro S.M.R., Pinheiro-Sant’Ana H.M. (2013). “Araçá of Cerrado” from the Brazilian savannah: Physical characteristics, chemical composition, and content of carotenoids and vitamins. Fruits.

[94] Raseira M.C.B., Raseira A. (1996). Contribuição ao estudo do araçazeiro (Psidium cattleyanum).

[95] Silva N.A., Rodrigues E., Mercadante A.Z., Rosso V.V. (2014). Phenolic compounds and carotenoids from four fruits native from the Brazilian Atlantic forest. J. Agric. Food Chem..

[96] Schroeter H., Heiss C., Balzer J., Kleinbongard P., Keen C.L., Hollenberg N.K., Sies H., Kwik-Uribe C., Schmitz H.H., Kelm M. (2006). (−)-Epicatechin mediates beneficial effects of flavanol-rich cocoa on vascular function in humans. Proc. Natl. Acad. Sci. USA.

[97] Link A., Balaguer F., Goel A. (2010). Cancer chemoprevention by dietary polyphenols: Promising role for epigenetics. Biochem. Pharmacol..

[98] Moreira R.R., Martins G.Z., Magalhaes N.O., Almeida A.E., Pietro R.C., Silva F.A., Cicarelli R.M. (2013). *In vitro* trypanocidal activity of solamargine and extracts from *Solanum palinacanthum* and *Solanum lycocarpum* of Brazilian Cerrado. An. Acad. Bras. Cienc..

[99] Miranda M.A., Kuehn C.C., Cardoso J.F., Oliveira L.G., Magalhaes L.G., Tiossi R.F., Rodrigues V., Zucolloto S., Prado J.C., McChesney J.D. (2013). Immunomodulatory effect of the alkaloidic extract of *Solanum lycocarpum* fruits in mice infected with *Schistosoma mansoni*. Exp. Parasitol..

[100] Yoshikawa M., Nakamura S., Ozaki K., Kumahara A., Morikawa T., Matsuda H. (2007). Structures of steroidal alkaloid oligoglycosides, robeneosides A and B, and antidiabetogenic constituents from the Brazilian medicinal plant *Solanum lycocarpum*. J. Nat. Prod..

[101] Qiu S., Sun H., Zhang A.H., Xu H.Y., Yan G.L., Han Y., Wang X.J. (2014). Natural alkaloids: Basic aspects, biological roles, and future perspectives. Chin. J. Nat. Med..

[102] Blankemeyer J.T., McWilliams M.L., Rayburn J.R., Weissenberg M., Friedman M. (1998). Developmental toxicology of solamargine and solasonine glycoalkaloids in frog embryos. Food Chem. Toxicol..

[103] Tiossi R.F., Da Costa J.C., Miranda M.A., Praca F.S., McChesney J.D., Bentley M.V., Bastos J.K. (2014). *In vitro* and *in vivo* evaluation of the delivery of topical formulations containing glycoalkaloids of *Solanum lycocarpum* fruits. Eur. J. Pharm. Biopharm..

[104] Farina F., Piassi F.G., Moyses M.R., Bazzolli D.M., Bissoli N.D.S. (2010). Glycemic and urinary volume responses in diabetic mellitus rats treated with *Solanum lycocarpum*. Appl. Physiol. Nutr. Metab..

[105] Vieira G., Ferreira P.M., Matos L.G., Ferreira E.C., Rodovalho W., Ferri P.H., Ferreira H.D., Costa E.A. (2003). Anti-infiammatory effect of *Solanum lycocarpum* fruits. Phytother. Res..

[106] Hall C.A., Hobby T., Cipollini M. (2006). Efficacy and mechanisms of α-solasonine-and α-solamargine-induced cytolysis on two strains of *Trypanosoma cruzi*. J. Chem. Ecol..

[107] Miranda M.A., Magalhaes L.G., Tiossi R.F., Kuehn C.C., Oliveira L.G., Rodrigues V., McChesney J.D., Bastos J.K. (2012). Evaluation of the schistosomicidal activity of the steroidal alkaloids from *Solanum lycocarpum* fruits. Parasitol. Res..

[108] Chataing B., Buitrago C.N., Concepcion J.L., Usubillaga A. (1996). Estudio clínico de la efectividad de extractos alcaloideos obtenidos de los frutos del *Solanun americanum* Miller sobre el herpes simplex herpes zoster y herpes genitalis. Rev. Fac. Farm..

[109] Pinto F.C.L., Uchoa D.E.A., Silveira E.R., Pessoa O.D.L., Braz-Filho R., Silva F.M., Theodoro P.N.E.T., Espíndola L.S. (2011). Antifungal glycoalkaloids, flavonoids and other chemical constituents of *Solanum asperum*. Quim. Nova.

[110] Fewell A.M., Roddick J.G., Weissenberg M. (1994). Interactions between the glycoalkaloids solasonine and solamargine in relation to inhibition of fungal growth. Phytochemistry.

[111] Lee K.-R., Kozukue N., Han J.-S., Park J.-H., Chang E.-Y., Baek E.-J., Chang J.-S., Friedman M. (2004). Glycoalkaloids and metabolites inhibit the growth of human colon (HT29) and liver (HepG2) cancer cells. J. Agric. Food Chem..

[112] Kuo K.-W., Hsu S.-H., Li Y.-P., Lin W.-L., Liu L.-F., Chang L.-C., Lin C.-C., Lin C.-N., Sheu H.-M. (2000). Anticancer activity evaluation of the *Solanum* glycoalkaloid solamargine: Triggering apoptosis in human hepatoma cells. Biochem. Pharmacol..

[113] Daunter B., Cham B.E. (1990). Solasodine glycosides. *In vitro* preferential cytotoxicity for human cancer cells. Cancer Lett..

[114] Munari C., de Oliveira P., Campos J., Martins S., Da Costa J., Bastos J., Tavares D. (2014). Antiproliferative activity of *Solanum lycocarpum* alkaloidic extract and their constituents, solamargine and solasonine, in tumor cell lines. J. Nat. Med..

[115] Punjabi S., Cook L.J., Kersey P., Marks R., Cerio R. (2008). Solasodine glycoalkaloids: A novel topical therapy for basal cell carcinoma. A double-blind, randomized, placebo-controlled, parallel group, multicenter study. Int. J. Dermatol..

[116] Bosco J., Soares K.T., Aguiar-Filho S.P., Barros R.V. (2000). A Cultura da Cajazeira.

[117] Tiburski J.H., Rosenthal A., Deliza R., de Oliveira Godoy R.L., Pacheco S. (2011). Nutritional properties of yellow mombin (*Spondias mombin* L.) pulp. Food Res. Int..

[118] Wang S., Melnyk J.P., Tsao R., Marcone M.F. (2011). How natural dietary antioxidants in fruits, vegetables and legumes promote vascular health. Food Res. Int..

[119] Roesler R., Malta L.G., Carrasco L.C., Holanda R.B., Sousa C.A.S., Pastore G.M. (2007). Antioxidant activity of cerrado fruits. Ciênc. Tecnol. Aliment..

[120] Roesler R., Catharino R.R., Malta L.G., Eberlin M.N., Pastore G. (2007). Antioxidant activity of *Annona crassiflora*: Characterization of major components by electrospray ionization mass spectrometry. Food Chem..

[121] Roesler R., Malta L.G., Carrasco L.C., Pastore G. (2006). Evaluation of the antioxidant properties of the Brazilian Cerrado fruit *Annona crassiflora* (Araticum). J. Food Sci..

[122] Coimbra M.C., Jorge N. (2011). Proximate composition of guariroba (*Syagrus oleracea*), jerivá (*Syagrus romanzoffiana*) and macaúba (*Acrocomia aculeata*) palm fruits. Food Res. Int..

[123] Duarte W.F., Dias D.R., Oliveira J.M., Teixeira J.A., de Almeida e Silva J.B., Schwan R.F. (2010). Characterization of different fruit wines made from cacao, cupuassu, gabiroba, jaboticaba and umbu. LWT Food Sci. Technol..

[124] Mateo J.J., Jimenez M. (2000). Monoterpenes in grape juice and wines. J. Chromatogr. A.

